# Translation of the Fear Avoidance Beliefs Questionnaire Into Hausa Language

**DOI:** 10.5539/gjhs.v7n3p116

**Published:** 2014-11-17

**Authors:** Bashir Kaka, Omoyemi O. Ogwumike, Opeyemi A. Idowu, Adesola C. Odole, Anas M. Saidu, Henrietta O. Fawole, Maryam Ibrahim

**Affiliations:** 1 Department of Physiotherapy, College of Health Science, Bayero University, Kano, Nigeria; 2 Department of Physiotherapy, College of Medicine, University of Ibadan, Ibadan, Nigeria; 3 Department of Physiotherapy, School of Basic Medical Sciences, College of Medicine, University of Benin, Benin-City, Nigeria; 4 Department of Nigerian Languages, Bayero University, Kano, Nigeria

**Keywords:** fear avoidance beliefs, Hausa, Validity, neck pain, patients, reliability, FABQ

## Abstract

**Background:**

Self-report measures of fear-avoidance beliefs are widely used in clinical practice and research. To date there is no Hausa version of the Fear Avoidance Beliefs Questionnaire (FABQ). This is important as the Hausa language is a widely spoken language in West Africa.

**Objectives:**

The purpose of this study was to translate and validate the Hausa version of the FABQ in patients with non-specific neck pain.

**Methods:**

Two independent bilingual Hausa translators translated the English version of the FABQ into Hausa which was thereafter back translated by one independent bilingual translator. A professional expert panel revised the translations to produce a consensus version. The psychometric testing of the final translated instrument was investigated by surveying 54 Hausa speaking patients with chronic non-specific neck pain. Cross-sectional construct validity was evaluated by comparing Hausa Fear Avoidance Beliefs Questionnaire (FABQ-H) with the English version of the FABQ. Internal consistency of the FABQ-H was examined by Cronbach alpha by comparing the scores between the FABQ-H and its subscales. Test-retest reliability was evaluated by administering the Hausa version twice.

**Results:**

The translated Hausa version of FABQ proved to be acceptable. The FABQ-H showed strong correlations (r=0.94, p=0.000) with the original English version. There was also high internal consistency between the FABQ-H and its subscales (physical activity component-α=0.88, p=0.000 and work component- α=0.94, p= 0.000). The FABQ-H also showed a high test-retest reliability (intra-class correlation coefficient =0.98).

**Conclusion:**

The FABQ-H demonstrated excellent psychometric properties similar to other existing versions. The FABQ-H is recommended for clinical practice.

## 1. Introduction

Neck pain can be defined as a subjective unpleasant sensory experience which manifests as fatigue, tension or pain with or without radiation to the shoulder, upper extremities or head (Siivola et al., 2002). It is a major public health concern associated with significant disability (Hogg-Johnson et al., 2008). Most people will experience neck pain at some points in their life although; it would not constitute disability (Hoy, Protani, De, & Buchbinder, 2010). Many researchers have attempted to classify neck pain and different methods have been proposed (Borghouts, Koes, & Bouter, 1998; Cote, Cassidy, & Carrol, 2003). The most widely accepted is the “diagnostic triage” which classifies neck pain patients as either (a) serious spinal pathology; (b) neurological involvement, or (c) non-specific neck pain (Kirchhoff et al., 2006; Klaber-Moffet & McLean, 2006). Of these three, non-specific neck pain accounts for the majority of complaints (Bertozzi et al., 2013).

Non-specific neck pain (NSNP) is pain (with or without radiation) with no specific objective underlying pathology (Binder, 2007). Many structures in the neck and surrounding regions such as muscles, joints, ligaments, intervertebral disc and neural structures may be sources of NSNP (Borghouts et al., 1998). Non-specific neck pain is perpetuated by physical, psychological, and individual related risk factors (Guzman et al., 2009). Studies have identified risk factors linked to the development of neck pain (McLean, May, Klaber-Moffet, Sharp, & Gardiner, 2010; Hogg-Johnson et al., 2008; Lee, Chiu, & Lam; 2007; Hill, Lewis, Sim, Hay, & Dziedzic, 2007). These include female gender, high job demands, smoking, psychosocial factors and co-morbid low back pain. Psychosocial factors are becoming more relevant in the onset, persistence and recurrence of neck pain (Ariens, Mechelen, Bongers, Bouter, & van der Wal, 2001). Fear avoidance beliefs’ are one such factor. According to Chou and Shekelle (2010), Fear avoidance beliefs describe patients’ worries that their pain/injury will be exacerbated by certain activities and these should be avoided. These beliefs often cause such individuals to avoid engaging in physical activities for fear of more pain or further injury (Chou & Shekelle, 2010; Leeuw et al., 2007). Fear avoidance beliefs if unchecked, can result in increased disability, work absenteeism, heavy reliance on medications and excessive use of medical services (Myhre et al., 2013; Mannion, Wieser, & Elfering, 2013; Linton & Andersson, 2000).

The Fear Avoidance Beliefs Questionnaire (FABQ) is a valid and reliable measurement tool for assessing fear avoidance beliefs in different populations (Rostami et al., 2014; Pei, Xia, & Yan, 2010; de Souza, Marinho, Siqueira, Maher, & Costa, 2008; Waddell et al., 1993). The FABQ is a 16-item instrument that assesses patients’ beliefs about the effects of physical activity and work on their musculoskeletal pain. Patients’ responses for each item are on a 7- point Likert scale (0 = completely disagree, 6 = completely agree). The original factor analysis revealed two subscales: the physical activity subscale (FABQpa) with 5 questions (maximum score = 24) and the work subscale (FABQw) with 11 questions (maximum score = 42). The score range is 0 to 96, with a higher value reflecting a higher degree of fear avoidance beliefs. A higher score indicates more strongly held fear avoidance beliefs. It takes about 10 minutes to complete (Waddell, Newton, Henderson, Somerville, & Main, 1993).

The translation and cultural adaptation of instruments is an internationally recognized method (Grassi-Oliveira, Stein, & Pezzi, 2006; Akinpelu, Maruf, & Adegoke, 2006; Maneesriwongul & Dixon, 2004; Li, Wang, & Shen, 2003). Instrument translation is a process by which an instrument is translated from one language to another. However, cross-cultural adaptation not only involves the initial translation process, but also synthesis, back translation, expert committee review, pre-testing of the consensus draft, cognitive debriefing interview (a way of getting feedback from respondents on the item-content, presentation, and clarity after pretesting) and psychometric evaluation. Cross-cultural adaptation usually encompasses a team of experts including the translators, researchers versed in clinimetrics (a way of evaluating signs, symptoms and laboratory findings with measures, indices, and other quantitative instruments) and health professionals who would normally use the instrument, as opposed to translation which requires only one or two translators. Cross-cultural adaptation is essential when the instrument is intended for use on a target population with cultural differences from that of the original version (Maher, Latimer, & Costa, 2007). The entire process of cultural adaptation is tailored towards achieving semantic and colloquial uniformity between the original and translated versions.

Nigeria is an ethnically diverse country of over 500 indigenous languages. However, Hausa, Igbo and Yoruba are the major ones (Babajide, 2001). The original FABQ was developed in the English language, the official language of communication in Nigeria. However, the majority of patients in Nigeria do not speak or write English (Akinpelu et al., 2006). It is therefore imperative to translate the FABQ into Hausa, a language which is not only spoken in Nigeria but also in some other African countries. Translating the FABQ into Hausa language may enhance the ease of use and accessibility by the Hausa speaking patient. Therefore, the objectives of this study were to translate the FABQ into Hausa language, culturally adapt it and to validate its use among Hausa-speaking non-specific neck pain patients.

## 2. Methods

### 2.1 Instrument

The FABQ developed by Waddell et al. (1993) investigates the fear-avoidance beliefs among patients in the clinical setting. The FABQ helps to predict individuals that have high pain avoidance behaviour. The FABQ consists of 2 subscales; the Physical Activity subscale (FABQpa) with items 1-5 and the Work subscale (FABQw) with items 6-16. Though not all items contribute to the score for each subscale participants are still expected to respond to all items. This is because none of the items were omitted during the initial validation of the scale. Each subscale is graded separately by totalling the responses of the scale items (0–6 for each item). For scoring purposes, only items 2,3,4 and 5 of the physical activity subscale (24 possible points) and only items 6, 7, 9, 10, 11, 12, and 15 of the work subscale (42 possible points) are scored. The total FABQ score is obtained by adding both subscale scores.

### 2.2 Translation Procedure

The recommended procedures for translating self-report measures were followed to translate the FABQ into Hausa language (Terwee et al., 2007; Beaton, Bombardier, Guilleman, & Ferraz, 2000). Two expert linguists proficient in both English and Hausa languages (and whose first language is Hausa) independently translated the FABQ into the Hausa language. One of the translators had a prior knowledge of the concepts being examined in the questionnaire. The other translator who neither worked in a medical institution nor had a medical background was uninformed about the concepts being examined. Each translator produced a written report of the translation they completed highlighting challenging phrases and uncertainties. They also summarized their rationale for their choice of words. Thereafter, both translators met to develop a reconciled version from both translations. A third linguist who was not involved in the original process of translation back-translated the questionnaire to English language.

A panel of professional experts comprising two experienced physiotherapists (involved in instrument development, translation and validation studies), the translators (forward and backward) and one of the researchers involved with this study (a native Hausa speaker) revised the back translation. The consensus Hausa translated version of the FABQ was then administered to seven patients with non-specific neck pain. Thereafter, the patients participated in a cognitive debriefing interview. All the patients reported that they understood all the items and that there was no imprecision in the questionnaire. Concurrent presentations of the English and the Hausa versions of the FABQ are shown in Table 1 while the complete version of the translated FABQ is presented in the appendix.

**Table 1:** Simultaneous presentation of English and Hausa version of the FABQ

1. My pain was caused by physical activity	1. Na samu ciwon wuya ne sanadiyyar wasar motsa jiki
2. Physical activity makes my pain worse	2. Wasar motsa jiki na sa ciwon wuyana ya qazanta
3. Physical activity might harm my neck.	3. Wasar motsa jiki zai illata mun wuya
4. I should not do physical activities which (might) make my pain worse.	4. Bai kamata in yi wasannin motsa jikin da (ka iya) qarfafa ciwo na ba
5. I cannot do physical activities which (might) make my pain worse.	5. Ba zan iya aiwatar da wasannin motsa jiki da (ka iya) qarfafa ciwo na ba
6. My pain was caused by my work or by an accident at work.	6. A dalilin haxari a wajen aiki ne sanadiyyar gamuwa ta da ciwon wuya
7. My work aggravated my pain.	7. Aikina kan kan haifar mun da tsananin ciwon wuya
8. I have a claim for compensation for my pain.	8. Na tura da da’awar ramuwar diyya dangane da ciwon wuyana
9. My work is too heavy for me.	9. Aikina ya yi mun nauyi sosai
10. My work makes or would make my pain worse.	10. Aikina zai sa zafin ciwon wuyana ya zamanto mafi muni
11. My work might harm my neck.	11. Aikina zai iya lahanta mun wuya
12. I should not do my regular work with my present pain.	12. Bai dace in yi ayyukan yau da kullum ba duba da ciwon da ke addabata
13. I cannot do my normal work with my present pain.	13. Ba zan iya ayyukan yau da kullum ba a yanayin ciwon da nake ciki
14. I cannot do my normal work until my pain is treated.	14. Ba zan iya ayyukan yau da kullum har sai an mun maganin ciwon wuyana

### 2.3 Validity and Reliability

According to the “Quality criteria for measurement properties of health status questionnaires”, it is essential to recruit no less than 50 participants for construct validity, reliability, and ceiling/floor effects analyses (Terwee et al., 2007). Therefore, 54 patients with chronic non-specific neck pain, who were bilingual in English and Hausa languages and aged 18 years and above were purposively recruited for the validation of the translated instrument. Individuals aged below 18 years and with co-morbidities that may have influenced overall wellbeing (such as sickle cell anemia, HIV/AIDS) and pregnant women with non-specific neck pain were excluded from the study. Participants were recruited from two tertiary health facilities (Aminu Kano Teaching Hospital and National Orthopedic Hospital, Dala) in Kano, Northern Nigeria. Ethical approval (AKTH/MAC/SUB/12A/P-3/VI/1175 and NOHD/RET/ETHIC/60) was sought and obtained from ethical committees of both the Aminu Kano Teaching Hospital and the National Orthopaedic Hospital. The procedure of the study was explained to each participant and thereafter his/her informed written consent was obtained before participating. Information on socio demographic characteristics (age, sex, marital status and occupation) of participants and clinical history of non-specific neck pain were obtained through interview and hospital files.

Copies of the English (FABQ-E) and Hausa (FABQ-H) versions of FABQ were concurrently self-administered to participants to determine the cross-sectional construct validity of the FABQ-H. The FABQ-H and the Hausa translated version of the Visual Analogue Scale (VAS) were also given to the participants to determine the divergent validity of the FABQ-H. After a week, the FABQ-H was given to participants to complete again to determine the test-retest reliability of the instrument.

### 2.4 Data Analysis

Descriptive statistics were used to summarize the age and pain intensity of participants. Percentages were used to summarize age group, gender, tribe, marital status, educational status and occupation of the participants. Participants’ scores obtained on the FABQ-H and FABQ-E were analyzed using Spearman rank order correlation to demonstrate the cross-sectional construct validity of the Hausa version. Spearman rank correlation was also used to determine the divergent correlation between the FABQ-H and VAS. The intra-class correlation (ICC) was used to investigate the correlation between the scores obtained by participants on the FABQ-H at two different occasions (test – retest reliability). The internal consistency between the FABQ-H and each of its two subscales: the physical activity subscale (FABQpa-H) and the work subscale (FABQw-H) were calculated using the Cronbach’s alpha. The level of significance (α) was set at 0.05. Data was analyzed using SPSS version 16.

## 3. Results

The validation part of this study involved 54 patients with chronic NSNP. The participants’ ages ranged between 23-65 years (mean age 39.30±10.86 years). They comprised of 36 (66.7%) males and 18 (33.3%) females. Twenty four (44.4%) of the participants had education up to tertiary level, 17 (31.5%) had attended up to secondary school while 13 (24.1%) had attended up to primary school. Socio-demographic characteristics of participants are presented in Table 2.

**Table 2:** Socio-demographic characteristics of the participants

Variable	Frequency	Percentage (%)
**Age Group**		
20-29	6	11.1
30-39	23	42.6
40-49	12	22.2
50-59	10	18.5
60 and above	13	5.6
**Gender**		
Male	35	64.8
Female	19.0	35.2
**Tribe**		
Hausa/Fulani	51	94.4
Others	3	5.6
**Marital Status**		
Married	47	87
Single	7	13
**Educational Status**		
Completed primary school	14	25.9
Completed secondary school	16	29.6
Completed tertiary education	24	44.4
**Occupation**		
Unemployed	21	38.9
Employed	18	33.3
Business	15	27.8

The mean scores of the participants on the FABQ-E and FABQ-H were 35.06±11.55 and 33.69±11.32 respectively. The mean pain intensity score of participants was 5.81±1.36. There was a significant positive correlation in the FABQw (r=0.92, p=0.000), FABQpa (r=0.94, p=0.000) and total FABQ (r=0.94, p=0.000) scores between the English and Hausa translated versions of FABQ. The correlation matrix in the subscale scores of the FABQ between the English and Hausa translated versions are shown in Table 3. Retesting 54 of the patients with NSNP seven days after baseline assessment revealed ICC values for the FABQ-H and its two subscales as: FABQ-H 0.98 (95% confidence interval [CI] 0.96-0.99); FABQpa-H 0.94 (95% CI 0.89-0.96) and FABQw-H 0.97 (95% CI 0.94-0.98). There was a positive significant correlation between the total scores on FABQ-H and each of FABQpa-H (α=0.88, p=0.000) and FABQw-H (α=0.94, p=0.000). Furthermore, there was a significant correlation between FABQw-H and FABQpa-H (α=0.67, p=0.000). Results also showed a weak correlation (α=0.40, p=0.003) between FABQ-H and pain intensity, as measured by the VAS.

**Table 3:** Correlation between English Version and Hausa Version of Fear Avoidance Beliefs Questionnaire using Spearman Rho Correlation

English	Mean ±SD	Hausa	Mean ±SD	R	p-value
FABQpa-E	16.65±5.17	FABQpa-H	16.30±5.14	0.94	.000**
FABQw-E	18.40±7.33	FABQw-H	17.39±7.20	0.92	.000**

*indicating significance correlation at p = 0.01. Key: FABQpa-E: Physical Activity subscale of the English version of Fear Avoidance Behaviour Questionnaire. FABQw-E: Work subscale of the English version of Fear Avoidance Behaviour Questionnaire. FABQpa-H: Physical Activity subscale of the Hausa version of Fear Avoidance Behaviour Questionnaire. FABQw-H: Work subscale of the Hausa version of Fear Avoidance Behaviour Questionnaire.

The FABQ-H had no ceiling/floor effects as less than 15% of participants had the minimum possible scores (n=1, 1.8%) in each of FABQpa-H and FABQw-H. None of the patients achieved the maximum possible scores in both subdomains of FABQ-H. All of the questions on the translated version were well accepted. The questionnaires were completed in 8±2 minutes. No missing responses or multiple answers were found and there were no reported problems with comprehension.

Here are some of the things other patients have told us about their pain. For each statement please circle the number from 0 (Completely disagree) to 6 (Completely agree) to indicate how much physical activities such as bending, lifting, walking or driving affect or would affect your neck pain.

The following statements are about how your normal work affects or would affect your neck pain.

## 4. Discussion

To the best of our knowledge, this is the first study to translate, culturally adapt and validate the FABQ for Hausa speaking patients with NSNP. Hausa is one of the three major Nigerian languages in a multi–ethnic country of over 500 indigenous languages (Babajide, 2001). There are over 50 million native speakers and 15 million non-native speakers of Hausa language in Northern Nigeria, the Republic of Niger, Northern Cameroun and Ghana (National African Language Resources Centre, 2010). Majority of the participants in this study speak Hausa as their first language.

The results of this study indicate that the FABQ-H is a reliable and valid instrument for measuring fear avoidance beliefs in Hausa-speaking patients with NSNP. It is also easily understandable and quick to complete, making it appropriate for use in routine health care. Clarity, reliability, cross-sectional construct validity and internal consistency of the FABQ-H are comparable to those of the original British version (Waddell et al., 1993). Two advantages come to the fore as a result of this work. Firstly, the FABQ-H is comparable to other internationally translated versions. Secondly, both the original and translated versions of the scale are comparable as a single instrument, without increasing the number of outcome measures.

In this study, FABQ-H demonstrated good test-retest reliability when the instrument was administered to participants a week apart. The ICC scores fell within the excellent reliability band of 0.70 to 0.95. This is concomitant with the validation studies of other translated versions: Persian [FABQpa: 0.802, FABQw: 0.808 and FABQ total: 0.805] (Rostami et al., 2014); Chinese [FABQpa: 0.78, FABQw: 0.84 and FABQ total: 0.85] (Pei, Xia, & Yan, 2010); Brazilian-Portuguese [FABQpa: 0.97, FABQw: 0.86 and FABQ total: 0.98] (de Souza et al., 2008), French [FABQpa: 0.72 and FABQw: 0.88] (Chaory et al., 2004).

The scores obtained on the FABQ-H correlated significantly with those on the FABQ-E. This indicates that the Hausa version measures the same construct as the English version. The correlation coefficient of 0.94 between the FABQ-H and FABQ-E found in this study falls within the excellent reliability band (0.70–0.95) for construct validity (Terwee et al., 2007). The Cronbach’s alpha values between the FABQ-H and each of FABQw-H and FABQpa-H, and also between FABQw-H and FABQpa-H indicate that the translated version is internally consistent. The Cronbach’s alpha of the two sub-domains of the FABQ-H ranged between 0.65 and 0.94. These values are similar to values reported by several studies on validity of other translations of the FABQ (Pei et al., 2010; de Souza et al., 2008; Grotle, Brox & Vollestad, 2006; Kovacs et al., 2006). To our knowledge, there is no “gold standard” measure of fear-avoidance and therefore it is not possible to assess criterion validity. However, the high correlations between the FABQ-H (and its subscales) and the weak correlation between FABQ-H and pain intensity, as measured by the VAS, provide satisfactory evidence for the construct validity of the instrument. It is not unanticipated that the latter correlation coefficient is low, since the FABQ and the VAS measure divergent constructs. It is however recommended that such validity should be explored with other scales such as the Neck Disability Index and Tampa scale of Kinesiophobia.

More studies are needed to investigate the relationship between self-reported fear avoidance beliefs, as measured by a questionnaire, and functional performance in patients with musculoskeletal pain, especially patients with NSNP. Also, this validation was done among patients with chronic NSNP, thus there is a need to further test the psychometric properties of the Hausa version among patients with acute and sub-acute symptoms and with other musculoskeletal conditions.
